# Metachromatic leukodystrophy genotypes in The Netherlands reveal novel pathogenic *ARSA* variants in non-Caucasian patients

**DOI:** 10.1007/s10048-020-00621-6

**Published:** 2020-07-07

**Authors:** Shanice Beerepoot, Silvy J.M. van Dooren, Gajja S. Salomons, Jaap Jan Boelens, Edwin H. Jacobs, Marjo S. van der Knaap, André B.P. van Kuilenburg, Nicole I. Wolf

**Affiliations:** 1 Amsterdam Leukodystrophy Center, Department of Child Neurology, Emma Children’s Hospital, Amsterdam University Medical Center, VU University Amsterdam and Amsterdam Neuroscience, De Boelelaan, 1117 Amsterdam, The Netherlands; 2 Center for Translational Immunology, University Medical Center Utrecht, Utrecht, The Netherlands; 3 Department of Clinical Chemistry, Metabolic Unit, Amsterdam University Medical Center, VU University Amsterdam, and Amsterdam Neuroscience, Amsterdam, The Netherlands; 4 Department of Clinical Chemistry, Laboratory of Genetic Metabolic Diseases, Amsterdam University Medical Center, University of Amsterdam, Amsterdam Gastroenterology & Metabolism, Amsterdam, The Netherlands; 5 Department of Pediatrics, Stem Cell Transplant and Cellular Therapies, Memorial Sloan Kettering Cancer Center, New York, NY USA; 6 Department of Clinical Genetics, Erasmus University Medical Center, Rotterdam, The Netherlands; 7 Department of Functional Genomics, Center for Neurogenomics and Cognitive Research, VU University, Amsterdam, The Netherlands; 8 Amsterdam UMC, location VUmc, De Boelelaan 1118, 1081 HV Amsterdam, The Netherlands

**Keywords:** *ARSA* gene, Arylsulfatase A, Metachromatic leukodystrophy, Genetic association studies

## Abstract

Metachromatic leukodystrophy (MLD) is an autosomal recessively inherited sulfatide storage disease caused by deficient activity of the lysosomal enzyme arylsulfatase A (ASA). Genetic analysis of the *ARSA* gene is important in MLD diagnosis and screening of family members. In addition, more information on genotype prevalence will help interpreting MLD population differences between countries. In this study, we identified 31 different *ARSA* variants in the patient cohort (*n* = 67) of the Dutch expertise center for MLD. The most frequently found variant, c.1283C > T, p.(Pro428Leu), was present in 43 (64%) patients and resulted in a high prevalence of the juvenile MLD type (58%) in The Netherlands. Furthermore, we observed in five out of six patients with a non-Caucasian ethnic background previously unreported pathogenic *ARSA* variants. In total, we report ten novel variants including four missense, two nonsense, and two frameshift variants and one in-frame indel, which were all predicted to be disease causing in silico. In addition, one silent variant was found, c.1200C > T, that most likely resulted in erroneous exonic splicing, including partial skipping of exon 7. The c.1200C > T variant was inherited in cis with the pseudodeficiency allele c.1055A > G, p.(Asn352Ser) + ∗96A > G. With this study we provide a genetic base of the unique MLD phenotype distribution in The Netherlands. In addition, our study demonstrated the importance of genetic analysis in MLD diagnosis and the increased likelihood of unreported, pathogenic *ARSA* variants in patients with non-Caucasian ethnic backgrounds.

**Electronic supplementary material:**

The online version of this article (10.1007/s10048-020-00621-6) contains supplementary material, which is available to authorized users.

## Introduction

Metachromatic leukodystrophy (MLD, OMIM #250100) is an autosomal recessively inherited sulfatide storage disease caused by deficient activity of the lysosomal enzyme arylsulfatase A (ASA). The disease is characterized by progressive central and peripheral demyelination, resulting in severe neurological deterioration. The most prominent signs and symptoms are ataxia, spasticity, cognitive decline, behavioral disturbances, peripheral neuropathy, and eventually a severely disabled state with epilepsy, painful spasticity, loss of motor and communication skills, and premature death [1]. Based on the age of symptom onset, three main MLD phenotypes can be distinguished: late-infantile (< 30 months), juvenile (2.5–16 years), and adult (> 16 years) MLD, with often an extra distinction between early-juvenile (2.5–6 years) and late-juvenile (6–16 years) MLD patients. A severe phenotype with symptom onset at a younger age, faster disease progression and shorter life expectancy, is usually accompanied by lower levels of residual ASA activity. However, a close correlation between disease severity and residual ASA activity could not be established [2–4].

Residual ASA activity levels are (partially) dependent on the two functional types of pathogenic *ARSA* variants that could be present: those resulting in inactive ASA (0-alleles, r.0), including the common splice donor site variants c.465 + 1G > A (r.0) and c.1210 + 1G > A (r.0), and those resulting in some residual ASA activity (R-alleles), including the frequently found missense variants c.1283C > T, p.(Pro428Leu) and c.542T > G, p.(Ile181Ser) [5]. Carriers of one pathogenic *ARSA* variant typically have reduced ASA activity although far above the ASA activity range of MLD patients [4, 6]. Although determination of ASA activity is very useful, in combination with the clinical symptoms and genetic findings, to diagnose MLD, it is not able to distinguish the different MLD phenotypes. ASA activity levels (1) show considerable variability between patients with the same clinical phenotype, even within families [7, 8]; (2) can vary within individual patients at repeated testing due to inter-assay variability [7]; and (3) can be reduced within the range of MLD patients in healthy individuals carrying two copies of the pseudodeficiency (Pd) allele c.1055A > G, p.(Asn352Ser) + ∗96A > G [9]. Due to the high frequency of the Pd allele, MLD patients may also have one or two copies of this allele in addition to their pathogenic *ARSA* variants. Some *ARSA* variants have been found to inherit in cis with the Pd allele [6]. Therefore, accurate genetic analysis of the *ARSA* gene is necessary in MLD diagnosis, especially when screening of (pre-symptomatic) family members is indicated [4].

In this study, we report the prevalence of pathogenic *ARSA* variants and MLD phenotypes in the patient cohort of the Amsterdam Leukodystrophy Center, a Dutch nationwide expertise center.

## Patients and methods

### Patient data

In this retrospective study, approved by the institutional review board and with appropriate consent of patients/their guardians, we included 76 patients who were referred to the Amsterdam University Medical Center (Amsterdam UMC) with a confirmed diagnosis of MLD. We considered MLD to be confirmed when at least two different tests were compatible with the diagnosis: homozygosity or compound heterozygosity for two pathogenic *ARSA* variants, increased urinary sulfatide excretion and/or decreased ASA activity within the range of MLD patients analyzed according to the Baum assay or modified Baum assay either at 0 or 4 °C depending on the laboratory [10–12], or when genetic testing of an affected sibling identified the same pathogenic *ARSA* variants as the index patient. In all but two patients, *ARSA* was tested directly by Sanger sequencing. In these two patients, *ARSA* mutations were detected using next-generation sequencing techniques (whole exome sequencing (WES) in a patient with unexplained polyneuropathy (MLD-67) and WES-based testing of a leukodystrophy gene panel in a patient with unexplained brain white matter abnormalities (MLD-81)). For most patients, both parents were tested to confirm the presence of mutations on two different alleles.

Genetic and biochemical tests were performed in the metabolic laboratory of the Amsterdam UMC, in laboratories collaborating with the referring hospitals including the metabolic laboratories of the Erasmus University Medical Center (Rotterdam), Radboud University Medical Center (Nijmegen), and University Medical Center Groningen (Groningen), or at both locations. Results of genetic analysis of the *ARSA* gene, ASA activity, urinary sulfatide excretion, and data on ethnicity, sex, age of symptom onset, presenting symptoms, presence of affected siblings, and consanguinity of the parents were collected from patient records. Patients who had not undergone genetic testing were excluded from this study.

According to the age of symptom onset, patients were grouped into a late-infantile, early-juvenile, late-juvenile, and adult phenotype. Reference-corrected residual ASA activity levels were constructed by expressing the ASA activity level of each individual patient as a percentage of the mean and the lowest boundary of the used reference values and in number of standard deviations (s.d.) from the mean when available. These reference-corrected residual ASA activity levels were used to examine the correlation between MLD phenotype and mean residual ASA activity with the Spearman rank correlation test and the independent sample *t* test in RStudio (version 3.6.1).

### *ARSA* variants

**Fig. 1:**
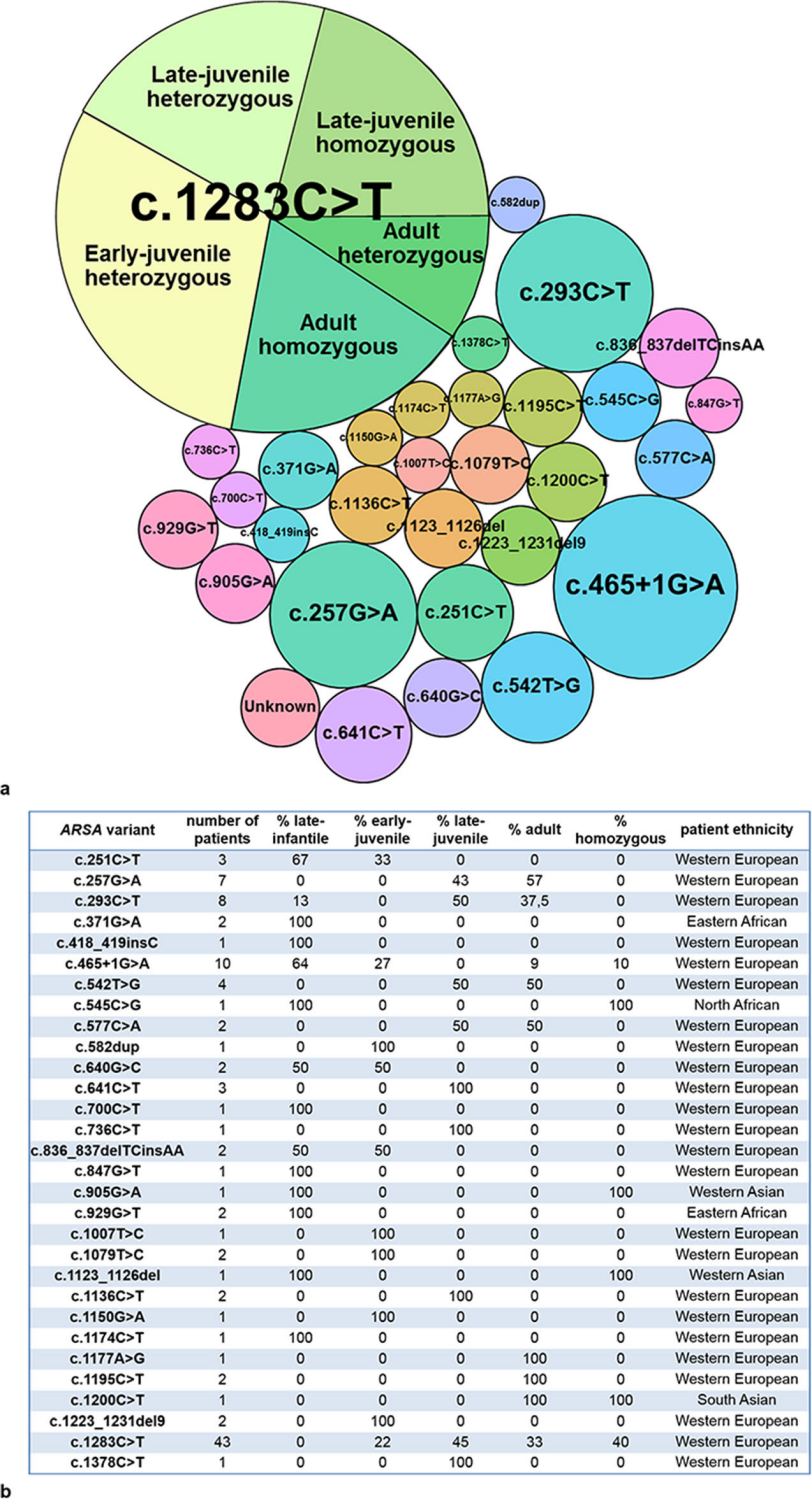
Comparative prevalence of all pathogenic *ARSA* variants in the patient cohort (GenBank accession number NM_000487.5) with their distribution of MLD type, zygosity, and ethnicity. **a** The size of the circle indicates the prevalence of the variant, and the color category of the circle corresponds the gene location shown in Fig. 2c. The most prevalent variant was c.1283C > T, p.(Pro428Leu), accounting for 45% of all the pathogenic *ARSA* variants, present in 43 out of 67 patients (64%). All patients carrying this variant had the early-juvenile (all heterozygous, *n* = 13), late-juvenile (heterozygous *n* = 9, homozygous n = 9), or adult MLD type (heterozygous *n* = 4, homozygous *n* = 8). The second and third most prevalent variant were c.465 + 1G > A (r.0) and c.293C > T, p.(Ser98Phe), accounting for respectively 8% and 6% of all pathogenic *ARSA* variants. **b** Table showing the number of patients and the distribution of MLD type, zygosity, and patient ethnicity for each of the pathogenic *ARSA* variants

**Fig. 2:**
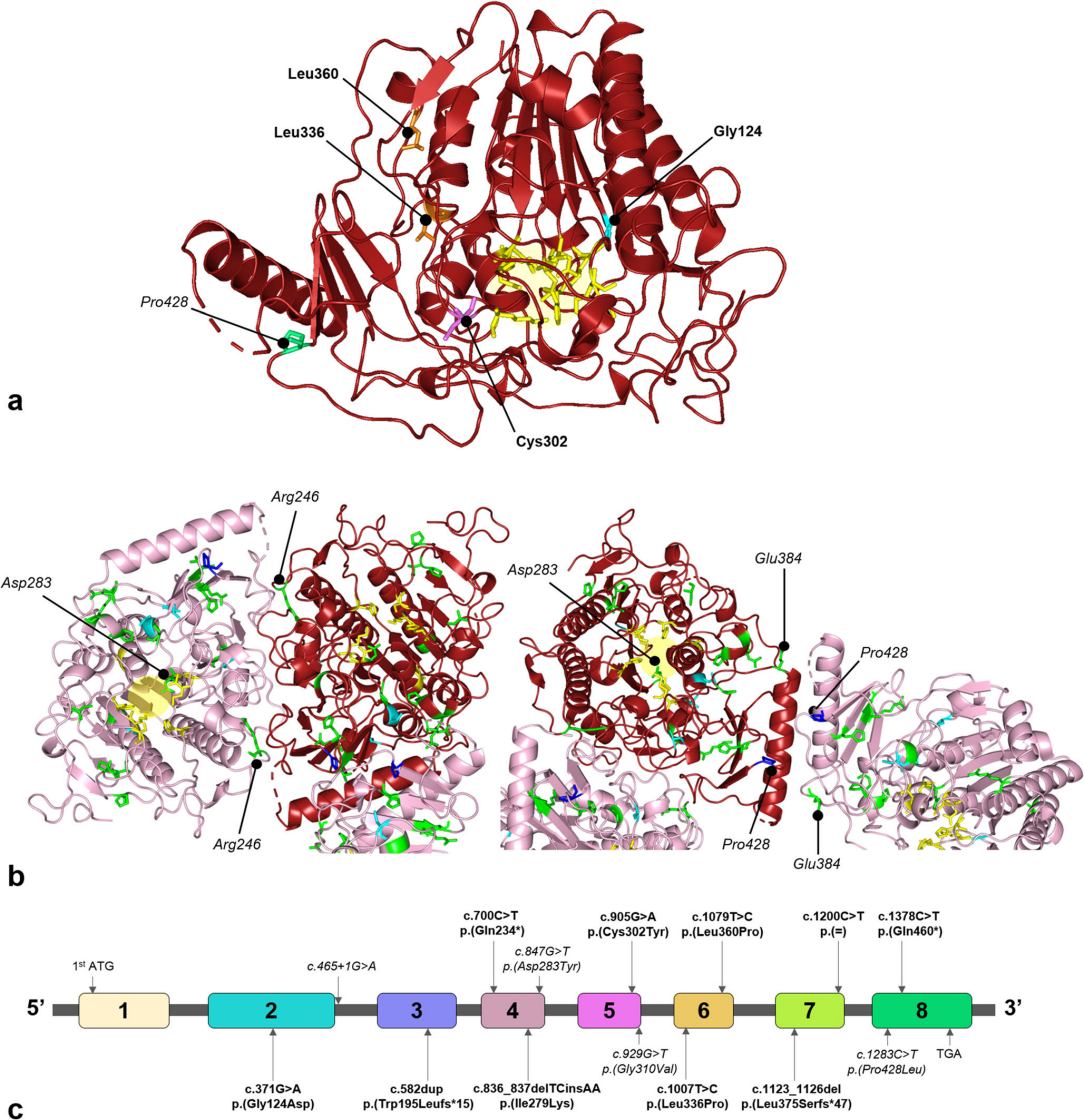
Pathogenic missense *ARSA* variants identified in this study. **a** 3D model of the ASA monomer showing novel pathogenic *ARSA* missense variants. Helices, β-sheets, and loops are shown as ribbons, arrows, and threads, respectively. The amino acid residues affected by the novel pathogenic *ARSA* missense variants (in bold) and c.1283C > T variant (in italic) are indicated with black spheres and highlighted in colors corresponding to their gene locations shown in Fig. 2c. The amino acid residues forming the catalytic site of ASA are highlighted in yellow. **b** 3D model of three subunits of the ASA octamer showing the location of all pathogenic missense *ARSA* variants identified in this study. Helices, β-sheets and loops are shown as ribbons, arrows and threads, respectively. Affected amino acid residues in the catalytic site or dimer interface are indicated with black spheres and highlighted in colors. The amino acid residues affected by previously reported variants are highlighted in green and those affected by the novel variants are highlighted in cyan. The common c.1283C > T variant is highlighted in blue. (c) The distribution of the novel pathogenic variants (in bold) throughout the *ARSA* gene. For the record, also the previously reported variants heterozygous with these novel variants are shown in italic. Numbered boxes represent the positions of the eight exons of the *ARSA* gene containing 509 amino acids (GenBank accession number NM_000487.5)

We reported all identified *ARSA* variants according to the current nomenclature guidelines (http://www.varnomen.hgvs.org) [13] and GenBank accession number NM_000487.5 (https://www.ncbi.nlm.nih.gov/nuccore/NM_000487.5) [14]. We consulted different databases, including 1000 Genome, Clinvar, and the Human Gene Mutation Database (HGMD), to investigate whether variants had previously been reported. For novel variants, we indicated whether they were missense, nonsense, silent or splice-site variants, or in-frame, frameshift deletions, insertions, duplications or indels. Furthermore, when available, data on parental presence of variants were analyzed to establish whether variants occurred de novo, whether they were biallelic or monoallelic, and whether they inherited in trans or cis with the Pd allele (when present). Potential pathogenicity of the variants was predicted in silico using MutationTaster, SIFT (Sorting Intolerant From Tolerant), PROVEAN (Protein Variant Effect Analyzer), and PolyPhen-2. For all variants, we investigated whether the affected amino acid was involved in the catalytic site or dimer interface of ASA based on the location within the crystal structures of human ASA monomer and octamer (PDB-ID: 1AUK) using PyMol [15, 16]. Fig. 1 was prepared with RStudio (version 3.6.1), using the packages “ggplot2” and “packcircles” [17]. Fig. 2 was made with PyMol based on the crystal structures of human ASA monomer and octamer (PDB-ID: 1AUK) [15, 16].

**Fig. 3:**
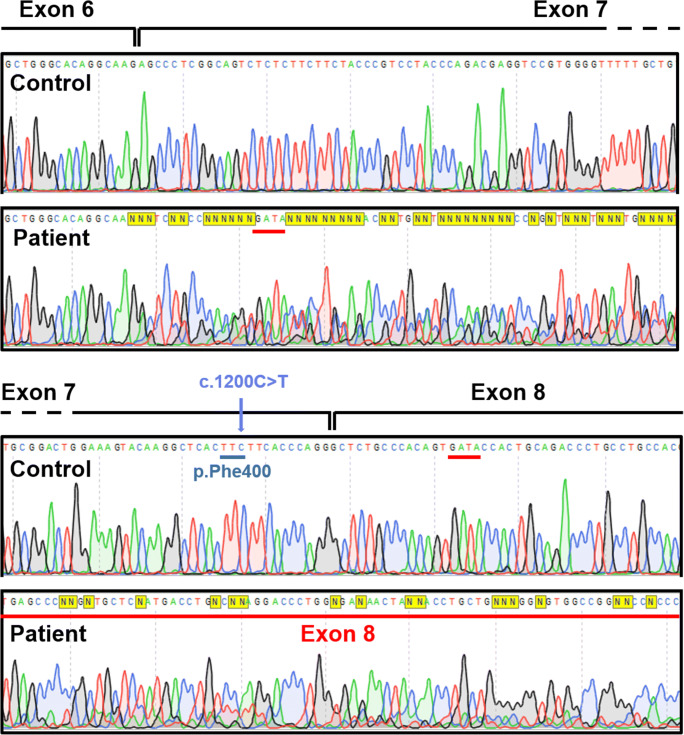
Demonstration of multiple splicing errors including partial skipping of exon 7 due to the c.1200C > T variant. *ARSA* mRNA was amplified by RT-PCR. The upper panel shows the cDNA sequence of a control, and the lower panel shows the sequence of the mutated cDNA fragment of the patient. The red bars indicate part of patient cDNA sequence corresponding with control cDNA sequence of exon 8

For one variant, c.1200C > T, p.(=), the effect on exonic splicing of *ARSA* pre-mRNA was analyzed by reverse transcriptase-polymerase chain reaction (RT-PCR) using the primers 5′-GTATCGGAAAGAGCCTGCTG-3′ and 5′-ACGTTATCAGGCACAAACCC-3′ and the PCR conditions as specified by the manufacturer. For this purpose, peripheral blood was collected in PAXgene collection tubes, and mRNA was extracted using a PAXgene Blood RNA Kit (#762164, PreAnalytiX, Qiagen/BD) according to the manufacturer’s instructions. Fig. 3, demonstrating the sequence of the mutated *ARSA* cDNA fragment of the patient compared to the *ARSA* cDNA sequence of a control, was made with SnapGene 4.2.9.

## Results

### Patient characteristics

Results of genetic analysis of the *ARSA* gene were available for 67 of the 76 MLD patients. The other nine patients had not undergone genetic testing and were therefore excluded from the study. Sixty-one of the 67 included patients had a Western European (Caucasian) ethnic background and six had a non-Caucasian ethnic background. The age of symptom onset ranged from 12 months to 36 years. Eleven patients (16%) had the late-infantile onset type of whom five had a non-Caucasian ethnic background, 39 patients (58%) had the juvenile onset type of whom fourteen patients with the early-juvenile (36%) and 25 patients with the late-juvenile (64%) phenotype, and seventeen patients (25%) had the adult onset type of MLD. An overview of individual patient characteristics is presented in Supplementary Table 1. Patients with later onset forms did not necessarily have higher residual ASA activity levels. Mean ASA activity per MLD phenotype, calculated as percentage of the mean and lowest boundary of the used reference values and in number of s.d. from the mean, are given in Supplementary Table 2. These reference-corrected means of residual ASA activity had also no or only a (very) weak correlation with the MLD phenotype (Spearman rank correlation coefficients of 0.11 (*p* = 0.41), 0.26 (*p* = 0.05), and 0.13 (*p* = 0.36), respectively), also when analyzed in homozygous MLD patients only (Spearman rank correlation coefficients of − 0.08 (*p* = 0.79), 0.10 (*p* = 0.71), and 0.00 (*p* = 1.00), respectively).

### Prevalence of *ARSA* variants

We identified a total of 31 different *ARSA* variants. The most frequent variant was c.1283C > T, p.(Pro428Leu), accounting for 44% of all pathogenic *ARSA* variants. Heterozygosity for this variant was observed in 26 patients (thirteen early-juvenile, nine late-juvenile, and four adult MLD patients), and homozygosity for the c.1283C > T variant was observed in seventeen patients (nine late-juvenile and eight adult MLD patients). In patients carrying the c.1283C > T variant, a strong correlation between MLD phenotype and reference-corrected means of residual ASA activity could again not be established (Spearman rank correlation coefficients of − 0.06 (*p* = 0.72; “mean corrected”), 0.06 (*p* = 0.74; “lowest boundary corrected”), and − 0.13 (*p* = 0.48; “s.d. corrected”)). Of all patients homozygous for the c.1283C > T variant, juvenile MLD patients showed even higher reference-corrected means of residual ASA activity compared to adult MLD patients (“mean corrected”: 11.4 vs 6.2, *p* = 0.003; “lowest boundary corrected”: 20.7 vs 14.9, *p* = 0.018; “s.d. corrected”: − 2.7 vs − 3.0, *p* = 0.314). All patients with the c.1283C > T variant had a Western European (Caucasian) ethnic background, and none of the patients homozygous for c.1283C > T became symptomatic before the age of 6 years. Importantly, none of the late-infantile MLD patients carried the c.1283C > T variant. The prevalence of each one of the other 30 pathogenic *ARSA* variants in our cohort ranged between 1 and 8%. The comparative prevalence of all *ARSA* variants in our cohort with their distribution of MLD type, zygosity, and ethnicity is shown in Fig. 1. In two siblings with MLD, only one pathogenic *ARSA* variant was detected. Finally, the Pd allele c.1055A > G, p.(Asn352Ser) + ∗96A > G was identified in 10 (15%) MLD patients. Heterozygosity for this variant was observed in eight Caucasian patients (one late-infantile, two early-juvenile, two late-juvenile, and three adult MLD patients), while homozygosity for this variant was observed in two non-Caucasian patients (one late-infantile and one late-juvenile MLD patient).

### Identification of ten novel variants

Five out of the six patients with a non-Caucasian ethnic background, two of whom were siblings, had a previously unreported pathogenic *ARSA* variant. In total, we identified ten novel pathogenic *ARSA* variants in thirteen patients from eleven unrelated families. An overview of clinical and genetic characteristics of these patients is presented in Table 1. Almost half (6/13) of these patients suffered from the late-infantile MLD form. Two late-infantile patients, both from consanguineous families, were homozygous for the novel variants (c.1123_1126del, p.(Leu375Serfs*47) or c.905G > A, p.(Cys302Tyr)). Except for these patients and one late-juvenile patient being homozygous for the novel variant c.1200C > T, p.(=), all other patients were compound heterozygous with a second, previously reported *ARSA* variant. Based on parental genetic data, we were able to establish that none of the variants occurred de novo and that the c.1200C > T variant was inherited in cis with the Pd allele c.1055A > G, p.(Asn352Ser) + ∗96A > G. The distribution of the novel variants over the *ARSA* gene and their location within the ASA protein are shown in Fig. 2.

**Table 1:** Overview of clinical characteristics of patients with their corresponding novel ARSA variants

Patient	Ethnicity	Sex	Type	Age at onset	Presenting signs and symptoms	Arylsulfatase A activity	Consanguinity of parents	Novel cDNA variant	Amino acid change	Type of mutation	Second cDNA variant	Amino acid change	Number of Pd allele variant in cis
MLD-25	South Asian	M	Late-juvenile	14 years	Cognitive decline and behavioral changes	3.3 nmol/h/mg (ref: 35–110)	Yes	*c.1200C > T*	*p.(=)*	Silent	*c.1200C > T*	*p.(=)*	2
MLD-26	Western European	M	Late-infantile	12 months	Ataxia and peripheral neuropathy	2.4 nmol/h/mg (ref: 35–110)	No	*c.700C > T*	p.(Gln234*)	Nonsense	c.847G > T	p.(Asp283Tyr)	No
MLD-29	Western European	F	Late-juvenile	6 years	Cognitive decline, ataxia and spasticity	3.0 nmol/h/mg (ref: 30–90)	No	*c.1378C > T*	p.(Gln460*)	Nonsense	c.1283C > T	p.(Pro428Leu)	No
MLD-36‡	Western European	M	Early-juvenile	5 years	Peripheral neuropathy and attention deficits	4.0 nmol/h/mg (ref: 35–110)	No	*c.1079 T > C*	*p.(Leu360Pro)*	Missense	c.1283C > T	p.(Pro428Leu)	No
MLD-37‡	Western European	F	Early-juvenile	± 5 years	Pre-symptomatic diagnosis	3.0 nmol/h/mg(ref: 35–110)	No	*c.1079 T > C*	*p.(Leu360Pro)*	Missense	c.1283C > T	p.(Pro428Leu)	No
MLD-38	Western European	M	Early-juvenile	4 years	Peripheral neuropathy, ataxia and spasticity	16.2 nmol/17 h/mg (ref: 81–300)	No	*c.582dup*	p.(Trp195Leufs*15)	Frameshift	c.1283C > T	p.(Pro428Leu)	No
MLD-45	Western European	F	Late-infantile	19 months	Peripheral neuropathy	4.0 nmol/17 h/mg(ref: 81–262)	No	*c.836_837delTCinsAA*	*p.(Ile279Lys)*	In-frame indel	c.465 + 1G > A	r.0	No
MLD-53	Western European	F	Early-juvenile	5 years	Peripheral neuropathy, ataxia and spasticity	1.0 nmol/h/mg (ref: 81–262)	No	*c.836_837delTCinsAA*	*p.(Ile279Lys)*	In-frame indel	c.1283C > T	p.(Pro428Leu)	No
MLD-57	Western Asian	F	Late-infantile	16 months	Peripheral neuropathy and spasticity	0.4 nmol/17 h/mg (ref: 45–260)	Yes	*c.1123_1126del*	p.(Leu375Serfs*47)	Frameshift	*c.1123_1126del*	p.(Leu375Serfs*47)	No
MLD-66	Western European	M	Early-juvenile	5 years	Peripheral neuropathy, ataxia and spasticity	6.8 nmol/h/mg (ref: 30–90)	No	*c.1007 T > C*	*p.(Leu336Pro)*	Missense	c.1283C > T	p.(Pro428Leu)	No
MLD-69‡	Eastern African	M	Late-infantile	24 months	Peripheral neuropathy, ataxia and spasticity	Undetectable(ref: 45–260)	No	*c.371G > A*	*p.(Gly124Asp)*	Missense	c.929G > T	p.(Gly310Val)	No
MLD-70‡	Eastern African	M	Late-infantile	± 24 months	Pre-symptomatic diagnosis	Undetectable(ref: 45–260)	No	*c.371G > A*	*p.(Gly124Asp)*	Missense	c.929G > T	p.(Gly310Val)	No
MLD-82	Western Asian	F	Late-infantile	24 months	Peripheral neuropathy and spasticity	3.0 nmol/h/mg(ref: 30–90)	Yes	*c.905G > A*	*p.(Cys302Tyr)*	Missense	*c.905G > A*	*p.(Cys302Tyr)*	No

Novel variants identified in this study are indicated in italic. *Abbreviations:* *‡* siblings, *F* female, *M* male, *nmol/h/mg* nanomole/h/mg protein, *ref.* reference values used in analysis, * stop codon, *fs* frameshift, *Pd* pseudodeficiency

### Characterization of novel variants

The ten novel variants included four missense variants, two nonsense variants, two frameshift variants (one single-base pair duplication and one four-base pair deletion), one in-frame indel, and one silent variant. None of these variants were present in 1000 Genome database, Clinvar, and HGMD. All novel variants were predicted to be disease causing by MutationTaster. All missense variants were predicted to be deleterious and probably damaging by SIFT, PROVEAN, and Polyphen-2. The in-frame indel variant (c.836_837delTCinsAA) was predicted to be possible damaging with HumDiv and HumVar scores of 0.78 and 0.87, respectively. The affected amino acids glycine on position 124 (c.371G > A), cysteine on position 302 (c.905G > A), and leucine on position 336 (c.1007 T > C) were highly conserved, while isoleucine on position 279 (c.836_837delTCinsAA) and leucine on position 360 (c.1079T > C) were moderately conserved. The silent c.1200C > T variant most likely created a cryptic splice-acceptor site in exon 7 (SpliceSiteFinder-like increase in the AG acceptor site at c.1208_1209 from 87.5 to 90.8), resulting in erroneous exonic splicing. RT-PCR analysis on *ARSA* mRNA extracted from the patient’s blood showed indeed multiple splicing errors including partial skipping of exon 7 (Fig. 3).

The sixth patient with a non-Caucasian ethnic background (MLD-34) had a North African ethnicity. Presenting signs were absence seizures, ataxia, and spasticity from age 12 months, and ASA activity was 7.0 nmol/h/mg (ref: –90 nmol/h/mg). She was homozygous for the c.545C > G, p.(Pro182Arg) variant and the Pd allele and had consanguineous parents. This variant was previously reported in one early-juvenile MLD patient heterozygous for this variant (second allele c.1283C > T) without data on ethnic background or the presence of the Pd allele [18].

## Discussion

The late-infantile MLD type is the most prevalent worldwide (48% of all patients) among MLD patients [3]. In contrast, we observed that in The Netherlands the juvenile type is much more common (58% of all patients). We showed that this is due to the high frequency of the c.1283C > T, p.(Pro428Leu) missense variant in Dutch MLD patients, a pathogenic *ARSA* variant that affects the stability of the ASA octamer by lowering the acidic pH [19]. The fact that this variant was not found in any late-infantile MLD patient and was found only in a heterozygous state in early-juvenile MLD patients confirms that this variant is associated with a later disease onset [3, 5, 20], however without strict correlation with relatively high residual ASA activity levels. The common observation that patients with later onset forms do not necessarily have higher residual ASA activity levels might be caused by multiple factors; e.g., *ARSA* variants might influence other ASA properties in addition to its activity in blood leukocytes. It is however important to consider that the ASA activity levels in this study were measured in different laboratories. Other possible explanations are therefore assay performance differences between laboratories or inter-assay variability.

In addition, we identified ten novel pathogenic *ARSA* variants. Two missense variants affected the same amino acid as missense variants previously reported as pathogenic. These two missense variants are the c.905G > A p.(Cys302Tyr) variant corresponding to the c.905G > T, p.(Cys302Phe) [21], and the c.371G > A, p.(Gly124Asp) variant corresponding to the c.370G > A, p.(Gly124Ser) and c.370G > T, p.(Gly124Cys) variants [22, 23]. Moreover, we identified the c.1200C > T variant as a potential disease-causing silent variant. Silent variants are often considered to be non–disease-causing since the amino acid sequence and subsequently protein structure and function are thought not to be altered [24]. However, the c.1200C > T variant seems to result in multiple splicing errors, and therefore, in agreement with segregation data in this family, to be a pathogenic *ARSA* variant. However, at this stage we cannot exclude the possibility that the observed altered pre-mRNA splicing of *ARSA* is due to an unknown intronic variant, in cis with the c.1200C > T variant. Taking into account the residual ASA activity and late-juvenile phenotype, it is likely that low levels of mRNA are spliced properly. Nevertheless, this is speculation and has not yet been investigated by analyzing multiple cDNA clones.

Remarkably, the presented group of patients with a novel *ARSA* variant had a much higher proportion of MLD patients with the late-infantile type (46%) and a non-Caucasian ethnicity (38%) compared to our full patient cohort (respectively 16% and 9%). This might be caused by the natural population prevalence of *ARSA* variants but might also be influenced by underdiagnosing and underreporting MLD patients with other ethnic backgrounds [25, 26]. This is a point of concern, considering global migration and in case genetic tests are employed that only test for common *ARSA* variants. False-negative MLD diagnosis could result in withholding treatment opportunities for otherwise eligible patients, especially since experimental therapies including gene therapy and intrathecal enzyme-replacement therapy are evolving. More phenotype information on pathogenic *ARSA* variants will also help interpreting results from the pilot newborn screening programs starting in parts of the USA and Europe, as prediction of the most likely MLD form will influence treatment decisions. Fortunately, novel *ARSA* variants in previously less frequently studied ethnicities are increasingly reported. A few examples are the c.847G > A, p.(Asp283Asn), c.853G > A, p.(Asp285Asn), and c.1031C > A, p.(Ala344Asp) variants in Sri Lanka [27]; the c.256C > G, p.(Arg86Gly), c.344 T > C, p.(Leu115Pro), and c.693C > A, p.(His231Gln) variants in respectively Jordan, Pakistan, and Tunisia [28–30]; and the c.1070G > T, p.(Gly357Val), c.585G > T, p.(Trp195Cys), c.849C > G, p.(Asp283Glu), and c.911A > G, p.(Lys304Arg) variants in Iran [31, 32]. Finally, Narayanan et al. recently reported 36 *ARSA* variants in MLD patients from India, and no less than sixteen of them were novel [33].

To conclude, with this study we provide a genetic base of the unique MLD phenotype distribution in The Netherlands. Our study also demonstrates the importance of genetic analyses in diagnosing and phenotyping MLD patients and stresses the need for sequencing the entire *ARSA* gene in patients with suspected MLD, instead of screening only for common *ARSA* variants. In case of variants of unknown significance and to confirm pathogenic variants, ASA activity in leukocytes or fibroblasts should be measured. Clinicians should be aware of unknown pathogenic *ARSA* variants in patients with various ethnic backgrounds.

## Electronic supplementary material

ESM 1 (PDF 138 kb)
