# Supplementary material for: Metachromatic leukodystrophy genotypes in The Netherlands reveal novel pathogenic ARSA variants in non-Caucasian patients
Source: Neurogenetics. 2020 Jul 7;21(4):289–99. doi: 10.1007/s10048-020-00621-6 (PMC7476914; doi:10.1007/s10048-020-00621-6)
Supplement: Supplementary file 1 — (PDF 138 kb) [file 10048_2020_621_MOESM1_ESM.pdf]

Supplementary Table 1: Overview of individual patient characteristics of all included patients

Patient	Ethnicity	cDNA variant 1	Amino acid change	cDNA variant 2	Amino acid change	Number of Pd alleles	Type	ASA activity (used reference values)	ASA activity expressed as percentage of reference mean	ASA activity expressed as percentage of lowest reference boundary	ASA activity expressed in s.d.
**MLD-1**	Western European	c.257G>A	p.(Arg86Gln)	c.293C>T	p.(Ser98Phe)	1	Adult	Very low	NA	NA	NA
**MLD-2**	Western European	c.257G>A	p.(Arg86Gln)	c.293C>T	p.(Ser98Phe)	1	Adult	4.8 nmol/h/mg (ref: 81 – 333)	2.3	5.9	-3.4
**MLD-4**	Western European	c.251C>T	p.(Pro84Leu)	c.1150G>A	p.(Glu384Lys)	0	Early-juvenile	32.0 nmol/h/mg (ref: 55 – 285)	18.8	58.2	-4.2
**MLD-5**	Western European	c.465+1G>A	r.0	c.1283C>T	p.(Pro428Leu)	0	Early-juvenile	9.8 nmol/h/mg (ref: 81 – 300)	5.1	12.1	-3.3
**MLD-6**	Western European	c.465+1G>A	r.0	c.1283C>T	p.(Pro428Leu)	0	Late-juvenile	10.4 nmol/h/mg (ref: 81 – 300)	5.5	12.8	-3.3
**MLD-8**	Western European	c.418_419insC	p.(His140fs)	c.640G>C	p.(Ala214Pro)	0	Late-infantile	8.6 nmol/h/mg (ref: 81 – 333)	4.2	10.6	-3.4
**MLD-9**	Western European	c.1283C>T	p.(Pro428Leu)	c.1283C>T	p.(Pro428Leu)	0	Late-juvenile	12.0 nmol/17h/mg (ref: 70 – 120)	12.6	17.1	-3.3
**MLD-12**	Western European	c.640G>C	p.(Ala214Pro)	c.1283C>T	p.(Pro428Leu)	0	Early-juvenile	6.0 nmol/h/mg (ref: 35 – 110)	8.3	17.1	-2.7
**MLD-13**	Western European	c.1283C>T	p.(Pro428Leu)	c.1283C>T	p.(Pro428Leu)	0	Adult	7.0 nmol/h/mg (ref: 35 – 110)	9.6	20.0	-2.6
**MLD-14**	Western European	c.1283C>T	p.(Pro428Leu)	c.1283C>T	p.(Pro428Leu)	0	Late-juvenile	6.0 nmol/h/mg (ref: 30 – 90)	10.0	20.0	-2.6
**MLD-15**	Western European	c.257G>A	p.(Arg86Gln)	c.293C>T	p.(Ser98Phe)	1	Adult	6.0 nmol/17h/mg (ref: 45 – 260)	3.9	13.3	-3.3
**MLD-16**	Western European	c.465+1G>A	r.0	c.542T>G	p.(Ile181Ser)	0	Late-juvenile	6.0 nmol/17h/mg (ref: 45 – 260)	3.9	13.3	-3.3
**MLD-17**	Western European	c.1223_1231del9	p.(Ser408_Thr410 del)	c.1283C>T	p.(Pro428Leu)	1	Early-juvenile	9.0 nmol/h/mg (ref: 30 – 90)	15.0	30.0	-2.4
**MLD-18**	Western European	c.1223_1231del9	p.(Ser408_Thr410 del)	c.1283C>T	p.(Pro428Leu)	1	Early-juvenile	10.0 nmol/h/mg (ref: 30 – 90)	16.7	33.3	-2.4
**MLD-21**	Western European	c.641C>T	p.(Ala214Val)	c.1283C>T	p.(Pro428Leu)	0	Late-juvenile	0.1 nmol/h/mg (ref: 9 – 30)	0.5	1.1	NA
**MLD-22**	Western European	c.641C>T	p.(Ala214Val)	c.1283C>T	p.(Pro428Leu)	0	Late-juvenile	0.1 nmol/h/mg (ref: 9 – 30)	0.5	1.1	NA
**MLD-23**	Western European	c.1283C>T	p.(Pro428Leu)	Unknown	Unknown	0	Early-juvenile	10.0 nmol/17h/mg (ref: 70 – 210)	7.1	14.3	NA
**MLD-24**	Western European	c.1283C>T	p.(Pro428Leu)	Unknown	Unknown	0	Early-juvenile	10.0 nmol/17h/mg (ref: 70 – 210)	7.1	14.3	NA
**MLD-25**	South Asian	c.1200C>T	p.(=)	c.1200C>T	p.(=)	2	Late-juvenile	3.3 nmol/h/mg (ref: 35 – 110)	4.6	9.4	-2.8
**MLD-26**	Western European	c.700C>T	p.(Gln234*)	c.847G>T	p.(Asp283Tyr)	1	Late-infantile	2.4 nmol/h/mg (ref: 35 – 110)	3.3	6.9	-2.8

**MLD-27**	Western European	c.257G>A	p.(Arg86Gln)	c.293C>T	p.(Ser98Phe)	1	Late-juvenile	11.0 nmol/17h/mg (ref: 70 – 210)	7.9	15.7	NA
**MLD-29**	Western European	c.1378C>T	p.(Gln460*)	c.1283C>T	p.(Pro428Leu)	0	Late-juvenile	3.0 nmol/h/mg (ref: 30 – 90)	5.0	10.0	-2.7
**MLD-30**	Western European	c.1283C>T	p.(Pro428Leu)	c.1283C>T	p.(Pro428Leu)	0	Adult	Very low	NA	NA	NA
**MLD-31**	Western European	c.1283C>T	p.(Pro428Leu)	c.1283C>T	p.(Pro428Leu)	0	Adult	Very low	NA	NA	NA
**MLD-32**	Western European	c.542T>G	p.(Ile181Ser)	c.1177A>G	p.(Thr393Gly)	0	Adult	5.0 nmol/17h/mg (ref: 45 – 260)	3.3	11.1	-3.3
**MLD-34**	North African	c.545C>G	p.(Pro182Arg)	c.545C>G	p.(Pro182Arg)	2	Late-infantile	7.0 nmol/h/mg (ref: 30 – 90)	11.7	23.3	-2.5
**MLD-35**	Western European	c.1283C>T	p.(Pro428Leu)	c.1283C>T	p.(Pro428Leu)	0	Late-juvenile	Very low	NA	NA	NA
**MLD-36**	Western European	c.1283C>T	p.(Pro428Leu)	c.1079T>C	p.(Leu360Pro)	0	Early-juvenile	4.0 nmol/h/mg (ref: 35 – 110)	5.5	11.4	-2.7
**MLD-37**	Western European	c.1283C>T	p.(Pro428Leu)	c.1079T>C	p.(Leu360Pro)	0	Early-juvenile	3.0 nmol/h/mg (ref: 35 – 110)	4.1	8.6	-2.8
**MLD-38**	Western European	c.582dup	p.(Trp195Leufs*15)	c.1283C>T	p.(Pro428Leu)	0	Early-juvenile	16.2 nmol/17h/mg (ref: 81 – 300)	8.5	20.0	-3.2
**MLD-39**	Western European	c.465+1G>A	r.0	c.1283C>T	p.(Pro428Leu)	0	Early-juvenile	6.8 nmol/h/mg (ref: 30 – 90)	11.3	22.7	-2.5
**MLD-40**	Western European	c.251C>T	p.(Pro84Leu)	c.293C>T	p.(Ser98Phe)	1	Late-infantile	5.0 nmol/h/mg (ref: 58 – 170)	4.4	8.6	-5.5
**MLD-41**	Western European	c.465+1G>A	r.0	c.542T>G	p.(Ile181Ser)	0	Adult	Very low	NA	NA	-5.5
**MLD-44**	Western European	c.1283C>T	p.(Pro428Leu)	c.1283C>T	p.(Pro428Leu)	0	Adult	4.0 nmol/h/mg (ref: 35 – 110)	5.5	11.4	-2.7
**MLD-45**	Western European	c.465+1G>A	r.0	c.836_837delTCinsAA	p.(Ile279Lys)	0	Late-infantile	4.0 nmol/17h/mg (ref: 81 – 262)	2.3	4.9	-5.5
**MLD-46**	Western European	c.1283C>T	p.(Pro428Leu)	c.1283C>T	p.(Pro428Leu)	0	Late-juvenile	5.0 nmol/17h/mg (ref: missing)	NA	NA	NA
**MLD-48**	Western European	c.1195C>T	p.(His399Tyr)	c.1283C>T	p.(Pro428Leu)	0	Adult	8.0 nmol/h/mg (ref: 35 – 110)	11.0	22.9	-2.6
**MLD-49**	Western European	c.1195C>T	p.(His399Tyr)	c.1283C>T	p.(Pro428Leu)	0	Adult	8.0 nmol/h/mg (ref: 35 – 110)	11.0	22.9	-2.6
**MLD-50**	Western European	c.251C>T	p.(Pro84Leu)	c.1174C>T	p.(Arg392Trp)	0	Late-infantile	Undetectable (ref: 30 – 90)	0.0	0.0	-2.9
**MLD-51**	Western European	c.1283C>T	p.(Pro428Leu)	c.1283C>T	p.(Pro428Leu)	0	Adult	12.0 nmol/h/mg (ref: 81 – 333)	5.8	14.8	-3.3
**MLD-53**	Western European	c.836_837delTCinsAA	p.(Ile279Lys)	c.1283C>T	p.(Pro428Leu)	0	Early-juvenile	1.0 nmol/h/mg (ref: 81 – 262)	0.6	1.2	-5.7
**MLD-54**	Western European	c.1283C>T	p.(Pro428Leu)	c.1283C>T	p.(Pro428Leu)	0	Late-juvenile	Very low	NA	NA	NA
**MLD-55**	Western European	c.1283C>T	p.(Pro428Leu)	c.1283C>T	p.(Pro428Leu)	0	Adult	6.0 nmol/h/mg (ref: 35 – 110)	8.3	17.1	-2.7
**MLD-56**	Western European	c.577C>A	p.(Pro193Leu)	c.1283C>T	p.(Pro428Leu)	0	Adult	15.0 nmol/h/mg (ref: 58 – 170)	13.2	25.9	-5.0

**MLD-57**	Western Asian	c.1123_1126del	p.(Leu375Serfs*47)	c.1123_1126del	p.(Leu375Serfs*47)	0	Late-infantile	0.4 nmol/17h/mg (ref: 45 – 260)	0.3	0.9	-3.4
**MLD-58**	Western European	c.1283C>T	p.(Pro428Leu)	c.1283C>T	p.(Pro428Leu)	0	Late-juvenile	6.7 nmol/h/mg (ref: 30 – 90)	11.2	22.3	-2.5
**MLD-59**	Western European	c.465+1G>A	r.0	c.465+1G>A	r.0	0	Late-infantile	10.1 nmol/h/mg (ref: 81 – 300)	5.3	12.5	-3.3
**MLD-60**	Western European	c.257G>A	p.(Arg86Gln)	c.293C>T	p.(Ser98Phe)	0	Late-juvenile	6.0 nmol/17h/mg (ref: 45 – 260)	3.9	13.3	-3.3
**MLD-61**	Western European	c.1283C>T	p.(Pro428Leu)	c.1283C>T	p.(Pro428Leu)	0	Late-juvenile	7.0 nmol/h/mg (ref: 30 – 90)	11.7	23.3	-2.5
**MLD-62**	Western European	c.1283C>T	p.(Pro428Leu)	c.1283C>T	p.(Pro428Leu)	0	Adult	6.0 nmol/17h/mg (ref: 45 – 260)	3.9	13.3	-3.3
**MLD-63**	Western European	c.1283C>T	p.(Pro428Leu)	c.1283C>T	p.(Pro428Leu)	0	Late-juvenile	Very low	NA	NA	NA
**MLD-64**	Western European	c.1136C>T	p.(Pro379Leu)	c.1283C>T	p.(Pro428Leu)	0	Late-juvenile	8.0 nmol/17h/mg (ref: 45 – 260)	5.3	17.8	-3.4
**MLD-65**	Western European	c.1136C>T	p.(Pro379Leu)	c.1283C>T	p.(Pro428Leu)	0	Late-juvenile	6.0 nmol/17h/mg (ref: 45 – 260)	3.9	13.3	-3.3
**MLD-66**	Western European	c.1283C>T	p.(Pro428Leu)	c.1007T>C	p.(Leu336Pro)	0	Early-juvenile	6.8 nmol/h/mg (ref: 30 – 90)	11.3	22.7	-2.5
**MLD-67**	Western European	c.577C>A	p.(Pro193Leu)	c.293C>T	p.(Ser98Phe)	0	Late-juvenile	2.9 nmol/17h/mg (ref: 45 – 260)	1.9	6.4	-3.3
**MLD-68**	Western European	c.1283C>T	p.(Pro428Leu)	c.1283C>T	p.(Pro428Leu)	0	Late-juvenile	15.0 nmol/17h/mg (ref: missing)	NA	NA	NA
**MLD-69**	Eastern African	c.371G>A	p.(Gly124Asp)	c.929G>T	p.(Gly310Val)	0	Late-infantile	Undetectable (ref: 45 – 260)	0.0	0.0	-3.4
**MLD-70**	Eastern African	c.371G>A	p.(Gly124Asp)	c.929G>T	p.(Gly310Val)	0	Late-infantile	Undetectable (ref: 45 – 260)	0.0	0.0	-3.4
**MLD-71**	Western European	c.736C>T	p.(Arg246Cys)	c.1283C>T	p.(Pro428Leu)	0	Late-juvenile	Very low	NA	NA	NA
**MLD-72**	Western European	c.465+1G>A	r.0	c.1283C>T	p.(Pro428Leu)	0	Late-juvenile	4.0 nmol/17h/mg (ref: 45 – 260)	2.6	8.9	-3.3
**MLD-73**	Western European	c.465+1G>A	r.0	c.1283C>T	p.(Pro428Leu)	0	Early-juvenile	3.0 nmol/17h/mg (ref: 45 – 260)	2.0	6.7	-3.3
**MLD-74**	Western European	c.1283C>T	p.(Pro428Leu)	c.1283C>T	p.(Pro428Leu)	0	Adult	5.8 nmol/17h/mg (ref: 45 – 260)	3.8	12.9	-3.3
**MLD-76**	Western European	c.641C>T	p.(Ala214Val)	c.1283C>T	p.(Pro428Leu)	0	Late-juvenile	6.0 nmol/17h/mg (ref: 45 – 260)	3.9	13.3	-3.3
**MLD-80**	Western European	c.257G>A	p.(Arg86Gln)	c.293C>T	p.(Ser98Phe)	0	Late-juvenile	3.3 nmol/17h/mg (ref: 45 – 260)	2.2	7.3	-3.3
**MLD-81**	Western European	c.257G>A	p.(Arg86Gln)	c.1283C>T	p.(Pro428Leu)	0	Adult	4.0 nmol/17h/mg (ref: 45 – 260)	2.6	8.9	-3.3
**MLD-82**	Western Asian	c.905G>A	p.(Cys302Tyr)	c.905G>A	p.(Cys302Tyr)	0	Late-infantile	3.0 nmol/h/mg (ref: 30 – 90)	5.3	10.7	-2.7

Abbreviations in table: fs = frameshift, del = deletie, * = stop codon, Pd = pseudodeficiency, ASA = arylsulfatase A, nmol/h/mg = nanomole/hour/milligram protein, NA = not applicable, ref = reference values used in analysis

Supplementary Table 2: Original and reference-corrected means of ASA activity per MLD phenotype

MLD phenotype (patient number)	Mean of original values given for ASA activity levels (s.d.)	Mean ASA activity expressed as percentage of reference mean (s.d.)	Mean ASA activity expressed as percentage of lowest reference boundary (s.d.)	Mean ASA activity expressed in s.d. of the mean of reference values (s.d.)
**Late-infantile (11)**	3.7 (3.6)	3.3 (3.5)	7.1 (7.2)	-3.5 (1.0)
**Early-juvenile (14)**	9.1 (7.7)	8.7 (5.4)	19.5 (14.2)	-3.1 (0.9)
**Late-juvenile (18)**	5.7 (3.4)	5.4 (3.8)	12.6 (6.3)	-3.1 (0.3)
**Adult (13)**	7.0 (3.2)	6.5 (3.7)	15.4 (6.0)	-3.2 (0.6)

Abbreviations in table: ASA = arylsulfatase A, nmol/h/mg = nanomole/hour/milligram protein, s.d. = standard deviation
